# A retrospective study on the efficacy and safety of Endostar with chemotherapy in EGFR-TKI-resistant NSCLC

**DOI:** 10.1186/s12890-023-02705-z

**Published:** 2023-11-11

**Authors:** Bing Han, Yanrong Kang, Haiji Wang, Jian Wang, Rong Shen, Shuai Liu, Lu Lu, Zhigang Sun, Nan Zhang

**Affiliations:** 1 Department of Obstetrics and Gynecology, Qilu Hospital of Shandong University, Shandong, 250013 China; 2 Department of Breast Center, Central Hospital, Shandong First Medical University, Jinan, Shandong 250013 China; 3 Department of Oncology, Yantai Hospital of Traditional Chinese Medicine, Yantai, Shandong 264001 China; 4 Department of Radiation Oncology, The Affiliated Hospital of Qingdao University, Qingdao, Shandong 266000 China; 5 Department of Medical Oncology, Qilu Hospital of Shandong University, Shandong, 250013 China; 6 Department of Chemotherapy, Shandong Provincial Hospital, Shandong First Medical University, Shandong, 250021 China; 7 Department of Thoracic Surgery, Central Hospital, Shandong First Medical University, Jinan, Shandong 250013 China

**Keywords:** Endostar, Chemotherapy, EGFR mutation, Resistance, Lung cancer

## Abstract

**Background:**

Endostar is a strong angiogenesis inhibitor that is effective in treating non-small cell lung cancer (NSCLC), but the effect of Endostar in the treatment of patients with EGFR-TKI-resistant NSCLC remains unclear. We evaluated the clinical efficacy and safety of Endostar in EGFR-mutant NSCLC patients resistant to EGFR inhibition treatment.

**Methods:**

From January 1, 2016 to June 30, 2018, 68 patients were selected from the 4 institutions for the study. Patients with NSCLC received Endostar plus chemotherapy every 21-day cycle. Chemotherapy types included platinum-containing dual drugs and platinum-free single drugs. Endostar was administered by intermittent intravenous infusion or continuous microinfusion pump infusion. The overall response rate (ORR), disease control rate (DCR) and adverse events were analyzed. Survival of patients was also evaluated.

**Results:**

For all patients, the median progression-free survival (PFS) was 2.8 months, and the median overall survival (OS) was 14.2 months. PFS and OS in the Endostar pump continuous group were better than those in the Endostar intravenous infusion group. The disease control rate (DCR) was 79.4%. A total of 28 (41.2%) patients experienced varying grades of adverse events during treatment. No treatment-associated deaths were observed. The grade 3 treatment-emergent adverse events (TEAEs) were myelosuppression, weakness, and nausea/vomiting.

**Conclusions:**

Endostar was effective and well tolerated in advanced NSCLC patients. Endostar treatment showed promising survival results in EGFR-mutant NSCLC patients.

## Introduction

Lung cancer is by far the most common malignancy and the leading cause of cancer-related death worldwide [1]. Non-small cell lung cancer (NSCLC) is the main histological subtype of lung cancer [2]. Epidermal growth factor receptor (EGFR)-sensitizing mutations have been found to be oncogene drivers for NSCLC that responds to EGFR tyrosine kinase inhibitors (TKIs). It has been reported that EGFR mutation is an established prognostic and predictive biomarker in NSCLC treatment [3–5]. Most EGFR mutations harbor an exon 19 deletion (ex19del) or exon 21 L858R in NSCLC, both of which render cancer sensitive to EGFR TKIs. Several phase III studies showed that EGFR TKIs as first-line treatment can improve PFS in comparison with standard chemotherapy in NSCLC patients with EGFR mutations [6–9]. EGFR TKIs have been documented in the first-line therapy of untreated advanced NSCLC with EGFR mutations and have revealed survival benefits and excellent tolerability [10–12]. Although EGFR TKIs initially have an outstanding therapeutic effect, most cancers exhibit resistance to EGFR TKIs, which is inevitable. Therefore, development of combination of targeted therapies should be important for NSCLC treatment.

One potential target for EGFR mutation resistance in NSCLC is the vascular endothelial factor (VEGF) pathway. Tumour angiogenesis has been recognized as the key factor in tumour development and progression, which is regulated by VEGF. Preclinical studies have shown a significant increase in VEGFR-1 expression in EGFR TKI-resistant lung cancer cells [13]. Inhibition of angiogenesis has been demonstrated as a novel and effective approach for lung cancer treatment. Endostatin was identified by Folkman et al. in 1997 and contains the 20 kDa C-terminal fragment of collagen XVIII. Previous studies using the recombinant human endostatin developed in China, have shown that Endostar can inhibit the VEGF-induced tyrosine phosphorylation of KDR/Flk-1 (VEGFR-2) [14]. Furthermore, the strong antiangiogenic effects of endostar were associated with the VEGF pathway. In accordance with a phase III study comparing vinorelbine-cisplatin (NP) plus Endostar versus NP alone in advanced NSCLC patients, the China State Food and Drug Administration licenced Endostar plus NP was utilized as a first-line therapy to treat advanced NSCLC in China [15]. Subsequently, several studies have demonstrated the clinical safety and anti-tumor efficacy of endostar in NSCLC patients [16–19]. In the EGFR TKI-resistant NSCLC population, the efficacy of chemotherapy alone is limited, and combined therapies may be more efficacious [20]. However, efficacy data for Endostar in EGFR-mutated resistant NSCLC are still lacking. There are two delivery types (intermittent intravenous infusion or continuous microinfusion pump infusion) of Endostar using in the treatment of cancer patients. However, whether different administrations influence the therapeutic efficacy and clinical outcomes in patients is unclear.

In this study, we addressed the question of whether Endostar is truly effective in the treatment of EGFR-mutated resistant NSCLC. We retrospectively analyzed the Endostar treatment in EGFR-TKI-resistant NSCLC patients and its related clinical outcomes in a real-world practice.

## Materials and methods

### Patient characteristics

In total, 68 patients with EGFR TKI-resistant NSCLC were included between January 2016 and June 2018. The inclusion criteria were specified as follows: (1) histopathological diagnosis of NSCLC; (2) unresectable or recurrent lung cancer shown by CT or MRI; (3) patients with EGFR-TKI resistance who used Endostar combined with chemotherapy; (4) stage IIIB or IV (defined by the 8th edition TNM staging system); (5) Eastern Cooperative Oncology Group score ≤ 2; and (6) at least one measurable lesion according to Response Evaluation Criteria in Solid Tumours, Version 1.1(RECIST v1.1). The study was approved by the Ethics Committee of each hospital (NO. AF/SC-07/04.0), and informed consent was collected from all the patients were collected.

### Treatment

Endostar is administered by intermittent intravenous infusion or continuous microinfusion pump infusion. The dose of Endostar was determined by body surface area (BSA). Endostar (7.5 mg/m^2^/24 h) was given by intravenous infusion on Days 1–14 or by 24-h continuous microinfusion pump infusion for Days 1–3 of each 21-day cycle. The time and dosage of chemotherapy were implemented in accordance with CSCO and NCCN guidelines, allowing doctors to adjust the dosage based on the specific situation of the patient. A CT scan was used at the beginning of therapy and the response to therapy was evaluated every two cycles by RECIST v1.1. Clinical data were collected at baseline, including sex, age, performance status(PS), smoking status, the lines of therapy and EGFR mutation status. Safety was observed during the study period. The toxicity reaction was graded based on NCI CTCAE version 4.03. Treatment-related adverse events were reported as explicitly stated in the file through the physicians or in the laboratory data gained during Endostar treatment.

### Follow-up

PFS was defined as the time from the start of first dosing to intolerable toxicity or progressive disease. OS was defined as the time from the start of first dosing to death. PFS and OS were collected and were estimated by using the Kaplan–Meier method. Disease progression, stable disease, or partial response was defined radiographically, dependent on the central radiologist’s final interpretation. The statistical analysis was carried out using SPSS version 27 (IBM Corp., Armonk, NY, USA). A *P* value < 0.05 was accepted as statistically significant.

## Results

### Patient characteristics

Patients were enrolled between 2016 and 2018 in our province. We evaluated 68 NSCLC patients for the effectiveness and safety of Endostar treatment. Table 1 provides the demographic and baseline clinical characteristics of the NSCLC patients. Forty-six patients were included in the Endostar endostar microinfusion pump group, while 22 patients were included in the Endostar endostar intravenous infusion group. Forty-three patients were male, and 25 patients were female. Twenty-one patients were over 65 years old. A total of fifty-two patients had ECOG PS = 1. Twenty-three patients had the EGFR exon 19 del. Nineteen patients had the EGFR exon 21 L858R mutation. Eight patients had the EGFR exon 20 T790M mutation. Fifty-three patients were resistant to first-generation TKI drug treatment. Fifteen patients suffered third-generation EGFR TKI drug resistance. Twenty-six patients were treated with Endostar as the second-line therapy. Forty-two patients were treated with Endostar as a third line or more. The presence of liver metastases was observed in 6 (8.8%) patients, and 15 (22.1%) patients had bone metastasis in the study.

**Table 1:** Baseline clinical characteristics of patients

Characteristics	*N* = 68(%)
Gender, n(%)	
Male	43(63.2)
Female	25(36.8)
Age (years), n(%)	
< 65	47(69.1)
≥ 65	21(30.9)
ECOG PS, n(%)	
0	11(16.2)
1	52(76.5)
2	5(7.3)
Smoking status, n(%)	
Yes	29(42.6)
No	39(57.4)
EGFR mutation, n(%)	
Exon 19 del	23(33.8)
Exon 21 L858R	19(27.9)
Exon 20 T790M	8(11.8)
Unselected	18(26.5)
TKI resistance, n(%)	
First/second generation	53(77.9)
Third generation	15(22.1)
Treatment lines of Endostar, n(%)	
Second line	26(38.2)
Further line	42(61.8)
Endostar administration mode, n(%)	
Microinfusion pump	46(67.6)
Intravenous infusion	22(32.4)
Chemotherapy, n(%)	
Platinum containing dual drug	44(64.7)
Platinum free single drug	24(35.3)
Liver metastasis, n(%)	
Yes	6(8.8)
No	62(91.2)
Bone metastasis, n(%)	
Yes	15(22.1)
No	53(77.9)

*Abbreviations*: *ECOG PS* Eastern Cooperative Oncology Group performance status, *EGFR* epidermal growth factor receptor, *TKI* tyrosine kinase inhibitor

### The efficacy of Endostar combined with chemotherapy in patients with EGFR-TKI resistance

**Fig. 1:**
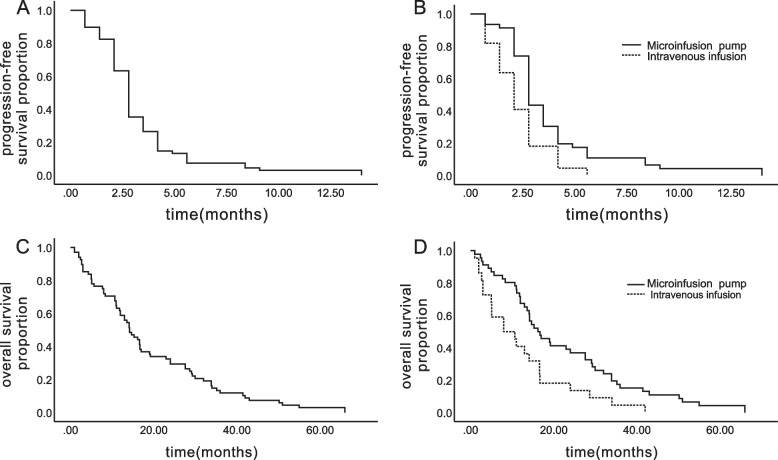
Kaplan–Meier analysis estimates of PFS (**A**, **B**) and OS (**C**, **D**). **A** PFS in all patients. **B** PFS in patients treated with endostar continuous microinfusion pump VS endostar intravenous infusion. **C** OS in all patients. **D** OS in patients treated with endostar pump continuous VS endostar intravenous infusion. Abbreviations: PFS, progression-free survival; OS, Overall Survival

The median PFS of all patients was 2.8 months (95% CI 2.515–3.085) (Fig. 1A). The median PFS was 2.8 months for patients in the Endostar microinfusion pump group (95% CI 2.471–3.129) and 2.1 months for patients in the intravenous infusion group (95% CI 1.467–2.733), which had a significant difference (*p* = 0.006) (Fig. 1B). Furthermore, the median OS of all patients was 14.2 months (95% CI 10.969–17.431) (Fig. 1C). The median OS was 16.2 months in the Endostar microinfusion pump group (95% CI 10.883–21.517) and 8.0 months in the intravenous infusion group (95% CI 1.220–14.780), which also had a significant difference (*p* = 0.007) (Fig. 1D). Multivariate analysis of all subgroups showed that route of administration was an independent prognostic factor for mPFS and mOS.

Compared with the microinfusion pump group, the risk ratio of disease progression and death ratio in the Endostar intravenous infusion group increased by 2.092 times (*P* = 0.013) and 2.454 times (*P* = 0.005), respectively. The overall survival of male patients was longer than female patients; however, it was not an independent prognostic factor of OS (Table 2). Among the patients who received Endostar combined with chemotherapy after EGFR-TKI failure, 7 patients achieved a partial response, and 47 patients were stable. The ORR was 10.3%, and the DCR was 79.4% among the patients (Table 3).

**Table 2:** Univariate and multivariate analysis of PFS and OS influencing factors

Characteristics	Univariate analysis		Multivariate analysis	
	HR (95% CI)	*P*	HR (95% CI)	*P*
PFS				
Gender	1.276(0.772–2.110)	0.342	1.059(0.511–2.194)	0.878
Age	1.310(0.775–2.213)	0.313	1.094(0.533–2.243)	0.807
ECOG PS	1.566(0.885–2.769)	0.123	1.506(0.772–2.938)	0.229
Smoking status	1.208(0.737–1.977)	0.454	1.712(0.841–3.485)	0.138
EGFR mutation	1.188(0.964–1.464)	0.107	1.181(0.912–1.530)	0.207
TKI resistance	1.148(0.643–2.049)	0.642	1.188(0.617–2.288)	0.607
Treatment lines of Endostar	1.453(0.888–2.377)	0.136	1.279(0.710–2.304)	0.413
Endostar administration mode	1.852(1.097–3.129)	0.021*	2.092(1.169–3.744)	0.013*
Chemotherapy	1.316(0.804–2.155)	0.275	1.151(0.648–2.045)	0.631
Liver metastasis	0.626(0.264–1.480)	0.286	0.561(0.221–1.422)	0.223
Bone metastasis	1.230(0.684–2.211)	0.490	1.593(0.788–3.220)	0.195
OS				
Gender	1.902(1.132–3.194)	0.015*	1.969(0.988–3.926)	0.054
Age	1.526(0.899–2.590)	0.117	1.206(0.625–2.328)	0.577
ECOG PS	1.481(0.726–3.024)	0.280	1.644(0.746–3.624)	0.217
Smoking status	1.397(0.836–2.336)	0.202	1.016(0.506–2.041)	0.963
EGFR mutation	1.165(0.953–1.424)	0.136	1.150(0.880–1.502)	0.307
TKI resistance	1.479(0.821–2.663)	0.192	1.859(0.946–3.653)	0.072
Treatment lines of Endostar	1.279(0.786–2.082)	0.322	0.935(0.501–1.744)	0.833
Endostar administration mode	2.037(1.202–3.453)	0.008*	2.454(1.302–4.625)	0.005*
Chemotherapy	1.008(0.619–1.641)	0.975	0.858(0.490–1.504)	0.594
Liver metastasis	0.898(0.393–2.105)	0.804	0.746(0.298–1.869)	0.532
Bone metastasis	1.016(0.567–1.821)	0.958	0.999(0.499–1.999)	0.997

^*^P < 0.05

**Table 3:** Efficacy analysis

	*N* = 68(%)
CR	0(0)
PR	7(10.3)
SD	47(69.1)
ORR	7(10.3)
DCR	54(79.4)

*Abbreviations*: *CR* complete response, *PR* partial response, *SD* stable disease, *ORR* objective response rate, *DCR* disease control rate

### Adverse events

The main adverse events of patients with the therapy are listed in Table 4. The most common adverse events were myelosuppression (29.4%), nausea/vomiting (14.7%), elevated transaminase (10.3%), weakness (7.4%), diarrhoea (2.9%), hematochezia (1.5%), elevated creatinine (1.5%), ST-T changes (2.9%), and arrhythmia (1.5%). Grade ≥ 3 adverse events included myelosuppression (13.2%), weakness (1.5%) and nausea/vomiting (2.9%). No clinically relevant grade ≥ 3 bleeding events occurred. No patients suffered drug-related deaths in this study.

**Table 4:** Drug-related adverse events

Adverse event	Any grade	≥ 3 grade
Any	28(41.2%)	12(17.6%)
Myelosuppression	20(29.4%)	9(13.2%)
Weakness	5(7.4%)	1(1.5%)
Nausea/vomiting	10(14.7%)	2(2.9%)
Diarrhea	2(2.9%)	0(0)
Hemoptysis	0(0)	0(0)
Hematochezia	1(1.5%)	0(0)
Elevated transaminase	7(10.3%)	0(0)
Elevated creatinine	1(1.5%)	0(0)
ST-T change	2(2.9%)	0(0)
Arrhythmia	1(1.5%)	0(0)
Fever	0(0)	0(0)
Alopecia	0(0)	0(0)
Allergy	0(0)	0(0)

## Discussion

Endostar is a novel angiogenesis inhibitor. Previous studies have assessed Endostar’s safety and efficacy [15–17, 21]. The Chinese Food and Drug Administration approved Endostar to treat NSCLC in 2005. Endostar combined with chemotherapy could refine OS and was well tolerated in patients with advanced NSCLC [22, 23]. Furthermore, Endostar combined with chemoradiotherapy for the treatment of advanced NSCLC could improve OS with tolerable toxicities [24]. In this study, we evaluated the clinical outcomes of Endostar as a second-line or higher-line therapy in patients with EGFR-TKI resistance.The purpose of our study was to evaluate the efficacy and safety of Endostar combined with chemotherapy. Our analyses demonstrated that Endostar provided a meaningful benefit in EGFR-mutant patients. To the best of our knowledge, this is the first study to explore the clinical significance of Endostar combined with chemotherapy for treating NSCLC patients with EGFR TKI resistance.

EGFR TKI therapy has displayed encouraging results in NSCLC patients with EGFR mutations [25], which is the first-line treatment option for advanced EGFR-mutated NSCLC. The clinical data suggested that use of 1st-generation EGFR TKIs (gefitinib and erlotinib) or 2nd-generation EGFR-TKIs as the first-line EGFR TKIs can adequately refine PFS and OS. However, EGFR TKI resistance, as the Achilles’ heel of targeted therapy in lung cancer, almost invariably limits the clinical efficacy of targeted. The five-year survival rate for EGFR-mutant metastatic lung cancer patients is approximately 15% [26]. There are different mechanisms of acquired resistance to 1st-generation and 2nd-generation EGFR-TKIs. The EGFR T790M mutation is the primary mechanism of 1st- and 2nd-generation EGFR-TKI resistance. The third-generation EGFR-TKIs, such as osimertinib, abivertinib, and nazartinib, which can target the T790M mutation, are satisfactory treatments. The mechanisms responsible for the 3rd generation EGFR-TKI resistance are complicated and still poorly understood, which include alternate pathway activation, target gene modification and histological or histologic transformation [27, 28]. Development of novel therapeutic strategies and rational combination regimens to reverse TKI resistance is urgently needed for promoting patient treatment and outcomes.

Many strategies have been developed to combat drug resistance by combining current therapies or by developing novel targeted agents. However, the median PFS of chemotherapy for EGFR-TKI-resistant NSCLC was only 4.0 months [20]. A previous study showed that PFS for patients with EGFR mutations was only 1.8 months with nivolumab monotherapy as first-line therapy [29]. To improve survival in EGFR TKI-resistant NSCLC patients, many scholars have explored the combination therapies. In reality, combined therapies may be more efficacious. Yoshihiro Hattori et al. reported the efficacy of bevacizumab plus chemotherapy in patients after failure of first-line EGFR-TKI inhibitor treatment. In their studies, the median PFS was 6.6 months, and the median OS was18.2 months [30]. In our current analyses, the median PFS was 2.8 months, and the median OS was 14.2 months. The difference between our and their results may be due to several reasons. First, Yoshihiro Hattori’s study was concentrated on the second line treatment after EGFR TKI resistance. In our study, we focused on the second-line, third-line or higher-line treatment after EGFR TKI resistance, and the survival benefit provided by the lateral lines of treatment was limited. Second, different pathways are inhibited by bevacizumab and Endostar in NSCLC. Moreover, atezolizumab plus bevacizumab plus chemotherapy showed significant improvements in PFS and OS in EGFR TKI-resistant patients; however, the incidence of adverse reactions was higher, especially for antiangiogenesis-related AEs such as hypertension and proteinuria [31, 32]. The results seem to indicate that this treatment might not be suitable for all patients with EGFR-TKI resistance.

In our study, the survival benefit of Endostar varied with different forms of administration. The median PFS was 2.8 months in the Endostar microinfusion pump group and 2.1 months in the intravenous infusion group. The median OS was 16.2 months in the Endostar microinfusion pump group and 8.0 months in the intravenous infusion group. Our finding is consistent with the previous research results [33]. A previous study showed that micropumps can maintain the effective blood concentration of Endostar for a long time [34]. Micropump 24-h continuous infusion of Endostar is more effective than routine infusion [35]. The half-life of Endostar in vivo is approximately 10 h [36]. Micropumps can deliver any drug suspension or solution at a constant rate for a prolonged period, thus maintaining the therapeutic concentration of Endostar [37]. Intravenous infusion significantly decreases the concentrations of Endostar, which may be the reason why patients in the Endostar microinfusion pump group experienced improved PFS compared to patients in the intravenous infusion group. In addition, we observed that sex was not an independent prognostic factor. The overall survival of male patients was better than that of female patients. This difference has also been reported in the E4599 study.

AEs have been recorded in the previous studies on Endostar [15–17]. There were no new AEs found in our current study. The most frequent grade ≥ 3 AEs were myelosuppression, weakness and nausea/vomiting when Endostar was used to treat patients with NSCLC after first-line EGFR-TKI therapy failure. The incidents of grade < 3 AEs were myelosuppression, weakness, nausea/vomiting, elevated transaminase, diarrhea, hematochezia, elevated creatinine, ST-T change, and arrhythmia. There were no class-related adverse effects of antiangiogenic treatment, such as venous thromboembolism, hypertension, or haemorrhage [38]. Based on these facts, there was no Endostar-related mortality. These results suggest that there are no safety-associated concerns regarding the use of Endostar combined with chemotherapy after EGFR TKI resistance.

Our study has several limitations. First, the major limitation is that this project is a retrospective study, and lacks randomization. We did not collect data on the duration of initial TKIs, dose delay and reductions, which could affect the outcomes of the study. Second, despite consolidating data from multiple institutions, the limited numbers of patients collected for the analyses was the major limitation of our current study. Due to the small sample size, the results cannot represent the whole population. Third, our study included a heterogeneous patient population who received Endostar in different treatment lines. The heterogeneity among different chemotherapies should also be mentioned. Finally, as a retrospective study, it was lacking in some important clinical information, such as data regarding chemotherapy cycles and radiotherapy. Despite these limitations, the current study utilized real-world data and provided a timely assessment of the dosing patterns of the newly approved treatment. These findings may be informative for real-world decision-making and future research in EGFR TKI-resistant NSCLC. Further large randomized controlled trials with long-term follow-up should be designed to assess the benefit of Endostar in EGFR TKI-resistant patients.

## Conclusions

In summary, to the best of our knowledge, this is the first study to evaluate the benefit of Endostar in NSCLC patients after EGFR TKI failure. Endostar plus chemotherapy provided clinical efficacy and safety after EGFR TKI resistance in the patients. Endostar may be a treatment option for NSCLC patients in EGFR TKI-mutation populations. Further research is warranted to determine the effects of Endostar in a large sample of patients.

## Data Availability

The datasets generated during and/or analysed during the current study are available from the corresponding author on reasonable request.

## Abbreviations

NSCLC: Non-small cell lung cancer
EGFR: Epidermal growth factor receptor
TKI: Tyrosine kinase inhibitor
VEGF: Vascular endothelial factor
PFS: Progression-free survival
OS: Overall survival
ORR: Objective response rate
DCR: Disease control rate
PS: Performance status
AE: Adverse event
